# Exosomes from Human Periapical Cyst-MSCs: Theranostic Application in Parkinson's Disease

**DOI:** 10.7150/ijms.41515

**Published:** 2020-02-24

**Authors:** Marco Tatullo, Benedetta Marrelli, Maria Josephine Zullo, Bruna Codispoti, Francesco Paduano, Caterina Benincasa, Francesco Fortunato, Salvatore Scacco, Barbara Zavan, Tiziana Cocco

**Affiliations:** 1 Marrelli Health - Tecnologica Research Institute, Biomedical Section, Street E. Fermi, Crotone, Italy; 2 Department of Therapeutic Dentistry, Sechenov University Russia, Moscow, Russia; 3 Department of Internal Medicine, Lausanne University Hospital, Lausanne, Switzerland; 4 Department of Neurological Sciences, University of Catanzaro “Magna Graecia”, Italy; 5 Department of Basic Medical Sciences, Neurosciences and Sense Organs, University of Bari “Aldo Moro”, Italy; 6 Department of Medical Sciences, University of Ferrara, Ferrara, Italy.

**Keywords:** exosomes, circadian rhythm, Parkinson's Disease

## Abstract

The scientific community continuously strives to get new disease models, to discover early markers or novel therapeutic approaches, improving the diagnosis and prognosis of several human pathologies. Parkinson's Disease (PD) is characterized by a long asymptomatic phase, characterized by a selective loss of dopaminergic neurons. Recently, the human Periapical Cyst-Mesenchymal Stem Cells (hPCy-MSCs) have been differentiated in functional dopaminergic neurons: such oral-derived MSCs and the hPCy-MSCs-derived exosomes may represent a strategic and useful *in vitro* study-model, as well as intriguing therapeutic carriers. Circadian rhythm (CR) alteration variously impacts on PD pathways: an interesting research target is represented by the analysis of the exosomes released by dopaminergic neurons, derived from neural-differentiated hPCy-MSCs, after having reproduced in-vitro PD-like conditions. This review aims to describe the crosstalk among some aspects of circadian rhythm related to the onset of PD and the exosomes released by cells of PD patients. More in detail: the first part of this article will describe the main characteristics of circadian rhythm and the involvement of the exosomes found to be effective in the pathogenesis of PD. Finally, the authors will suggest how those exosomes derived from dopaminergic neurons, obtained by oral-derived stem cells (hPCy-MSCs) may represent a smart model for the *in vitro* research on PD, to find new biomarkers, to test new drugs or, fatally, to find new pathways applicable in future therapeutic approaches.

## Introduction

Circadian rhythm is an endogenous process that, through complex cellular and molecular networks, regulates the organism's behavior following the sleep- wake cycle, and repeats approximately every 24hours. The circadian rhythm allows complex organisms to be able to sustain all such environmental changes that regularly occur during the light-dark cycles, thus impacting on alimentation, metabolisms, movement, and immunological reply to pathogens 1.

Clock genes commonly regulate the circadian rhythm: they are expressed by cells located in the suprachiasmatic nucleus (SCN), in the hypothalamus. These genes act as a regulatory system that modulates all the circadian mechanisms, working within the complex organisms 2. In mammals, the circadian clock follows a hierarchical organization: in fact, the suprachiasmatic nucleus is stimulated by light signals that are converted into electrical signals within the retina; such electrical information is then transduced to cells and organs, through the activation of the “secondary oscillators” working as local effectors in peripheral tissues 3.

The circadian system is self-sustainable, and it regularly works across the day under stable and physiological conditions; nevertheless, it may be impaired by those entraining cues called “zeitgebers” 1.

At the molecular level, several transcriptional “feedback loops” can regulate the secondary oscillators: this set of proteins may act as positive (CLOCK, BMAL1) or negative (CRY1-2, PER1-3) transcription factors, upon binding to E-box (like) enhancer elements. The alteration or loss of clock genes results in a consequent impairment or loss of circadian rhythm 4.

Altered circadian rhythms are also associated with several human metabolic diseases, including type-2 diabetes and cardiovascular diseases, which represent a significant risk to public health**.** The molecular signal transduction pathways that mediate between stress responses and peripheric organs rhythms are aimed to maintain metabolic homeostasis under different conditions 5.

Numerous evidences have demonstrated that there are close and multilevel interactions between neurodegenerative pathologies and the alteration of the circadian rhythm.

More in details, the circadian genes can impact on the tyrosine hydroxylase, the controlling enzyme aimed to modulate the dopamine synthesis; such pathway is regulated by the circadian locomotor cycle kaput (Clock) gene. Moreover, the circadian genes may also affect the dopaminergic activity in the ventral tegmental area of the brain, and dopamine is conversely able to regulate some Clock genes with a feedback mechanism 6. In fact, dopamine is able to upregulate the transcriptional activity of Clock/Bmal complex: this action is carried out by an increase of the phosphorylation of transcriptional coactivator cAMP-responsive element-binding proteins. Hood et al. reported that the rhythm of PER2 (period circadian regulator 2) expression could be modified by D2 (dopamine-2) receptor activation 7. Imbesi et al. found that D2 receptor agonists inhibited the expression of Clock and Per1 genes, while D1 receptor agonists enhanced the expression of Per1 and Clock genes 8.

On the other hand, Yujnovsky et al. reported a reduced transcription of the Per1 gene in D2R (dopamine D2 receptor)-null mice models 9. Dopamine alteration can also impair the proper rhythm during the prenatal stage in the circadian pacemaker SCN 10, 11. The light/dark alternation regulates the melatonin and the main mechanisms acting under the circadian rhythm: the melatonin acts as the main stimulator of the fetal SCN 12 and is also controlled by dopamine-receptor signaling 13.

This concise review aims to investigate the connection among some aspects of circadian rhythm related to the onset of Parkinson's Disease (PD) and the extracellular vesicles released in PD patients.

Finally, the authors will suggest how those exosomes derived from dopaminergic neurons, obtained by novel oral-derived stem cells may represent a smart model for the *in vitro* research on PD.

## Genetic, endogenous and environmental factors can impact PD onset and severity

The genetic pathway involved in PD pathogenesis depends on the type of genetic mutation. Currently, four genes are clearly involved in the autosomal dominant form of PD: the SNCA/PARK1- PARK4 gene, the LRRK2/PARK8 gene, the VPS35/ PARK17 gene, and the EIF4G1/PARK18 gene; on the other hand, PARK-1 and DJ-1 genes are typically involved in the autosomal recessive transmission. A combination of genetic predisposition and environmental factors is the most intriguing topic to understand the basis of PD early-onset; the chronic interaction with toxic substances in the living environment certainly increases the possibility to develop damages to the mitochondria, which are responsible for the increase of Reactive Oxygen Species (ROS) in the body, and which are closely associated with the production of misfolded α-Synuclein 14.

Genetic mutations in the genes that code for PARKIN and PINK-1 are closely associated with PD and mitochondria alterations: these genetic alterations may be triggered by several factors, and they represent a crucial step in the onset of several neurodegenerative pathologies. In fact, mitochondria play a key role in cell metabolism, so that an impaired activity of the mitochondrial complexes has been widely reported in the Substantia Nigra of PD patients. Mitochondria are extremely susceptible to high levels of oxidative stress, often induced by the presence of ROS that may increase in such cases where mitochondria complexes work badly.

It is well known that the formation of super-aggregates of misfolded and toxic accumulation of α-synuclein is pathognomonic for PD: a point mutation of the SNCA gene, coding for α-synuclein, is related to oxidative stress and to impaired activity of serotonergic neurotransmitters, a dysfunction that occurs before the clinical diagnosis of Parkinson's disease.

An abnormal accumulation of α-synuclein, due to genetic mutations of the related gene, can be cause and consequence of sleep-wake rhythm alterations. This correlation is not only a kind of side effect of PD symptomatology, but the sleep-wake rhythm alterations concretely influence the severity of PD, as a consequence of the primary alteration of the serotonergic system, in turn, associated to sleep dysfunction in PD 15.

## Circadian rhythm: a metronome able to modulate the content of the extracellular vesicles in PD patients

Circadian rhythm (CR) is a natural metronome regulating gene expression in mammalian and eukaryotic cells.

Exosomes have been reported to act in several processes: one of the most interesting is the RNA degradation 16, which is closely linked to the reduction of such proteins that plays a pivotal role in the peripheral control of those functions regulated by CR. The link between RNA degradation, CR and exosomes activity is closely related to the regulation of cell cycle, driving the early steps of cell development: the same cellular mechanism has been observed in both prokaryotic and eukaryotic, suggesting that this peculiar link has been conserved over the evolutionary development 17.

The exosomes contain several bioactive molecules closely related to the CR activity: this evidence increasingly promoted studies aimed to understand the real involvement of EVs in the synchronization between signals generated by the SNC and the peripheral effectors. It is biologically reasonable that exosomes could act as carriers of information across the intricate clock circuits 18.

CR is often related to the onset of several neurodegenerative diseases; in fact, the CR alteration seems to anticipate the clinical symptoms of cognitive and non-cognitive impairments 19. Exosomes could be considered as therapeutic tools, able to control and preserve the CR integrity: they could be potentially used for the treatment of such diseases linked to impairments of CR. Although there is contrasting evidence, the link between CR alteration, exosomes impairment and neurological diseases may be a consequence of multiple alterations of specific proteins production; moreover, the pathogenesis may be triggered by immunological dysfunctions 20.

In this context, recent studies have consistently demonstrated that exosomes secreted by PD neurons spread the α-synuclein (α-syn), a pathognomonic protein secreted by neurons representing the main component of Lewy's bodies; moreover, PD-related exosomes can increase neuroinflammation, thus playing a role in PD worsening 21.

PD develops its clinical signs starting from a massive degeneration of dopaminergic neurons (DA) located in the human brain. Intracellular proteins alteration is potentially involved in the common pathogenesis of PD: these alterations mainly involve endosomes and lysosomes 22. In this context, the secretion of extracellular vesicles (EVs), specifically the exosomes, is influenced by processes that modulate the protein synthesis: in PD, the EVs content represents a "snapshot" of the intracellular environment, selecting molecules acting as biomarkers in the local microenvironment 23. Exosomes and their content are typically involved in cell-to-cell communication, acting as shuttles of molecules that can influence target-cells with biomarkers (miRNAs, proteins, lipids). PD patients have been reported to have impaired expression of genes related to CR 24.

Moreover, PD patients have an altered secretion pattern of melatonin, especially PD, with a non-tremor-dominant phenotype. A potential explanation of the link among CR and PD onset could be partially related to the significant reduction of Period Circadian Regulator 1 and Brain and Muscle ARNT-Like 1 expression in leucocytes. Dopamine seems to have a central role in this mechanism: in fact, it can affect the BMAL1/CLOCK heterodimer activity, thus modulating one of the most strategic molecular players working in CR 25.

Recent studies have pointed out that neurons of PD patients are also subjected to several alterations of mitochondria and endoplasmic reticulum, combined with alterations in the proteosome-ubiquitin system, leading to the onset of such neurodegenerative disease. Moreover, PD patients frequently show severe dysfunctions in the mitochondrial metabolism of Ca_2+_. Specifically, Parkin is an E3 ubiquitin-protein ligase, able to modulate Ca_2+_ mitochondrial homeostasis: it is involved in the binding between the ubiquitin and the polypeptide-chains. On the other hand, it has been also demonstrated that the expression of mutant forms of Parkin might affect the structure and the physiology of endosomes and endo-lysosomal pathway: such mutations are peculiar of PD patients, which frequently show an increase in the releasing of exosomes 26.

PD patients have the typical loss of Parkin: this anomaly has been related to a decrease in endosomal tubulation and an increased formation of intraluminal vesicles, coupled with a greater release of exosomes 27. This impaired mechanism stimulates Parkin to regulate the Rab7 levels: in fact, Rab7 is highly expressed in Parkin-free cells, and it is also fatally associated with the increased production of exosomes, suggesting that Rab7 deregulation may be partially responsible for the endocytic phenotype observed in Parkin-deficient cells 28.

Mitochondrial damage in PD has been reported to be frequently related to the interaction between PINK1 and Parkin 29,30. PINK1 activates the phosphorylation of ubiquitin of the outer mitochondrial membrane: this phosphorylation activates Parkin, which consequently increases its ubiquitin ligase activity; as a consequence of this pathway, Parkin promotes the cellular autophagy and the formation of vesicles derived from damaged mitochondria 29. Parkin and PINK1 appear to be mutated in familial Parkinson's disease. Furthermore, Parkin and PINK1 inhibit the molecular mechanisms leading to the presentation of the mitochondrial antigen (MitAP) by blocking the transport of vesicles derived from mitochondria (MDV) 30.

This association among Parkin, PINK1, mitochondrial damage, and impaired production of exosomes after mitochondrial autophagy in PD cells strongly suggests that the studies based on exosomes from PD cell-models are a strategic way to carry out novel results on this severe neurodegenerative disease. A strategic future challenge would be to investigate whether CR alteration is able to influence the PD onset, or if it should be considered only a risk factor. In fact, impairment of CR impacts the expression of several genes, and it can locally trigger inflammatory responses, oxidative stress, and it can also impair the degradation of protein aggregates that play a pivotal role in neuroinflammation and neurodegeneration 31.

## The human Periapical-Cyst-Mesenchymal Stem Cells (hPCy-MSCs): a novel MSC line

Recent studies have shown that MSCs can also differentiate towards phenotypes of great clinical interest, such as neuron-like phenotype 32, 33.

MSCs residing in the oral cavity are commonly defined as "Oral Derived-MSCs" (ODSCs) 34: recently, the authors have discovered ODSCs even in inflamed periapical cysts 35; these cells have been characterized and called human Periapical-Cyst-Mesenchymal Stem Cells (hPCy-MSCs). Further studies demonstrated that such cells are a promising tool in regenerative medicine 36, 37.

HPCy-MSCs express a specific panel of surface markers, such as CD13, CD29, CD44, CD73, CD105, CD90 and STRO-1 38,39; moreover, these cells have high plasticity and they can easily differentiate towards adipose and cartilaginous cells, towards osteoblasts and neurons 40.

Immunofluorescence and cytofluorimetry analyses on hPCy-MSCs have found that they naively express the main neuronal markers, such as β-III tubulin, and the main astrocytes markers, such as glial fibrillary acidic protein (GFAP). Gene expression in hPCy-MSCs exposed to neurogenic culturing conditions showed a notable increase in neural markers: surprisingly, these cells preferentially differentiate towards dopaminergic neurons, already after 21 days of culture in the neuro-inductive medium 41.

The spontaneous commitment of these cells towards neuronal differentiation and in particular towards the formation of dopaminergic neurons raises interesting implications in the development of new therapies involving hPCy-MSCs, as could be the development of Parkinson's cell models to explore the biochemical pathways involved in the onset of the disease in an *in vitro* system.

The advantages of using hPCy-MSCs, compared with plasma samples, are related to the easy procedure for isolating MSCs from a tissue that would otherwise be eliminated (this is the key- concept of the so-called “waste medicine”): this main advantage is certainly not to be underestimated. In fact, the possibility of using a "biological waste", represented by each discarded tissue usable in reparative/ regenerative medicine, that becomes a rich source of stem cells represents an important opportunity to highly evaluate these cells in regenerative procedures 42.

On the other hand, hPCy-MSCs are abundant and easy to find within biological tissue, usually discharged: moreover, hPCy-MSCs have been demonstrated to have similar stemness properties of the well-known dental pulp stem cells, thus making them safe and reliable in tissue engineering.

The releasing of extracellular vesicles (EVs) content has been considered a strategy makes cells able to eliminate harmful compound; furthermore, recent researches have suggested that EVs act as a bridge among CR effectors and targets, just like a kind of messengers of eukaryotic cells. EVs are transporters of several biological molecules, and they are released in the extracellular environment, both in pathological and physiological conditions 43.

Typically, the exosome's content includes nucleic acids, cytokines, proteins, and enzymes, misfolded proteins 44.

Numerous studies carried out in the last years have confirmed the role of exosomes in modulating several neuronal activities: a pivotal role of these vesicles has been described in the synaptic activity among different neurons, confirming the commitment of these extracellular messengers 45.

An increased number of exosomes in the *substantia nigra* has been often related to several neurodegenerative diseases; in PD, scientists have observed an increased number of exosomes that mainly transport α-synuclein to target cells, inducing their apoptosis. This strategy may suggest that exosomes' content plays a key role in the worsening of clinical conditions suffered by patients with PD. In fact, the spreading of α-synuclein and its aggregates in the brain can be considered as one of the main steps towards the PD progression. On the other hand, some authors also reported that exosomes could be strategic actors for targeted therapy in PD patients, as they can act as a shuttle for the local drug-releasing and their content has been carefully investigated to discover new biomarkers for PD 46.

In the familiar PD, α-synuclein aggregates are typically observable and known as “Lewy bodies”. Interestingly, the overall α-synuclein-mRNA levels increase in PD mid-brain, but they do not change in the PD patient's frontal cortex. In this landscape, exosomes are the main actors in the releasing of biological molecules to target cells located in different areas of the brain. In fact, these small carriers can contribute to PD propagation, through spreading the misfolded α-synuclein from affected cells to normal cells 47.

In PD, the degeneration of dopaminergic neurons depends on the pathological accumulation of such misfolded and toxic forms of α-synuclein in the cytoplasm: neuron-derived exosomes are also able to carry α-synuclein toxic forms to astrocytes and microglia, so contributing to PD spreading also as a consequence of inflammatory mechanisms. In the light of the consistent impact of neuron-derived exosomes and their contents to PD, these markers can be the most useful in a strategy aimed to improve the early diagnosis and to find novel therapeutic strategies for PD in a cell-model, as we suggest in this review.

Dopaminergic neurons (DA) could be obtained after the exposition of hPCy-MSCs to neural-inductive medium; the isolation of exosomes from the conditioned culture medium is currently standardized 48. The characterization of the exosomes content could be obtained using electron microscopy, cytofluorimetry, and western blotting: it has been shown that exosomes carry a series of molecules from one cell to another; a strategic diagnostic approach could be based on the analysis of microRNAs in plasma and circulating exosomes. In fact, microRNAs are a class of RNA molecules that, due to their small size, can cross the blood-brain barrier and appear in the bloodstream so their role can be relevant for PD in the initial stages (asymptomatic) and the various phases of development 49,50.

The isolation of the exosomes from hPCy-MSCs will allow several analyses on miRNAs through microarrays and gene sequencing. The analysis of small-sized circulating exosome-derived micro-RNA could be usefully associated with nanotechnologies: this is important to improve the ability of novel smart nanomaterials to intercept the ultralow-molecular- weight biomolecules, so acting as a theranostic tool with high sensitivity and high specificity 51,52.

In this light, the isolation, purification, and characterization of those molecules enclosed in hPCy-MSCs-derived exosomes, and released in the culture medium, may provide important information on biomarkers usable for the early diagnosis and the monitoring of neurodegenerative diseases, with a special interest toward PD.

In this landscape, hPCy-MSCs-derived exosomes are a kind of experimental platform for analyzing and developing novel therapies, based on the study of molecules carried by these extracellular vesicles.

## Conclusions and future insights

As properly described in this review, several scientific pieces of evidence have clearly shown that impairments in the CR can play a pivotal role in the pathogenesis of neurodegenerative diseases. Altered expression of clock genes has been found in the peripheral blood cells of PD patients 25.

Exosomes contain and transport several types of molecules, including membrane proteins and cytosolic proteins, messenger RNAs, and ncRNAs (such as miRNAs): the ability to share information among neurons is mediated by such exosomes, released in the surrounding environment.

In this context, the characterization of the exosomes released in the culture medium of dopaminergic neurons differentiated from hPCy- MSCs could represent an interesting strategy.

We hypothesized that the hPCy-MSCs, properly differentiated into dopaminergic neurons, could be characterized by specific markers. Following the differentiation of hPCy-MSCs into neurosecretory dopaminergic neurons, the characterization of the exosomes may be performed both on neurons differentiated by hPCy-MSCs obtained from healthy subjects and by subjects affected by PD. The existence of a potential link among the alteration of circadian rhythm and the onset of PD has been proposed in the scientific literature. The genes involved in circadian rhythm are regulated by secreted molecules, embedded in extracellular vesicles; thus, the study of the secretory pattern of cells from healthy subjects and patients affected by PD could help the researchers to understand better those mechanisms that may correlate the circadian rhythm and the onset of PD. In this landscape, the possibility of using a new smart source of MSCs derived from the inflamed periapical cysts, that have previously and consistently showed a spontaneous tendency to neural differentiation, could represent an advantageous and free-of-biological- costs model for novel and highly repetitive *in vitro* investigations, to find new biomarkers, to test new drugs or, fatally, to find new pathways to use in novel therapeutic approaches.
